# The association between telomere length and blood lipids: a bidirectional two-sample Mendelian randomization study

**DOI:** 10.3389/fendo.2024.1338698

**Published:** 2024-05-28

**Authors:** Shengjie Yang, Xinyue Wang, Yujuan Li, Lijun Zhou, Gang Guo, Min Wu

**Affiliations:** ^1^Guang’an men Hospital, China Academy of Chinese Medical Sciences, Beijing, China; ^2^Qilu Hospital of Shandong University, Jinan, China

**Keywords:** telomere length, blood lipids, bidirectional two-sample Mendelian randomization, aging, dyslipidemia

## Abstract

**Background:**

Observational studies suggest an association between telomere length (TL) and blood lipid (BL) levels. Nevertheless, the causal connections between these two traits remain unclear. We aimed to elucidate whether genetically predicted TL is associated with BL levels via Mendelian randomization (MR) and vice versa.

**Methods:**

We obtained genetic instruments associated with TL, triglycerides (TG), low-density lipoprotein cholesterol (LDL-C), high-density lipoprotein cholesterol (HDL-C), apolipoprotein A-1 (ApoA-1) and apolipoprotein B (ApoB) from large-scale genome-wide association studies (GWASs). The causal relationships between TL and BL were investigated via bidirectional MR, multivariable MR and mediation analysis methods. The inverse variance weighted (IVW) method was employed as the principal methodology, complemented by several other estimators to enhance the robustness of the analysis.

**Results:**

In the forward MR analyses, we identified significant positive correlation between genetically predicted TL and the levels of TG (β=0.04, 95% confidence interval [CI]: 0.01 to 0.06, p = 0.003). In the reverse MR analysis, TG (β=0.02, 95% CI: 0.01 to 0.03, p = 0.004), LDL-C (β=0.03, 95% CI: 0.01 to 0.04, p = 0.001) and ApoB (β=0.03, 95% CI: 0.01 to 0.04, p = 9.71×10^–5^) were significantly positively associated with TL, although this relationship was not observed in the multivariate MR analysis. The mediation analysis via two-step MR showed no significant mediation effects acting through obesity-related phenotypes in analysis of TL with TG, while the effect of LDL-C on TL was partially mediated by body mass index (BMI) in the reverse direction, with mediated proportion of 12.83% (95% CI: 0.62% to 25.04%).

**Conclusions:**

Our study indicated that longer TL were associated with higher TG levels, while conversely, higher TG, LDL-C, and ApoB levels predicted longer TL, with BMI partially mediating these effects. Our findings present valuable insights into the development of preventive strategies and interventions that specifically target TL-related aging and age-related diseases.

## 1 Introduction

Telomeres are DNA-protein structures located at the terminal regions of chromosomes that play a crucial role in maintaining genomic stability and cellular integrity (1). Gradually shortening over time in most somatic tissues (2), telomere length (TL) is considered to be a biomarker of biological aging (3, 4). Moreover, TL is increasingly being recognized as a clinical indicator of age-related disease risk (5), including cardiovascular and cerebrovascular diseases, diabetes, cancer, and neurodegenerative disorders (6).

Blood lipids (BL) are fatty substances and apolipoproteins circulating in blood. Commonly measured BL traits include triglycerides (TG), low-density lipoprotein cholesterol (LDL-C), high-density lipoprotein cholesterol (HDL-C), apolipoprotein A-1 (ApoA-1) and apolipoprotein B (ApoB) (7, 8). Abnormal blood lipid levels are associated with various diseases. Previous studies have found that controlling BL levels can effectively reduce the risk of cardiovascular disease (9, 10), with LDL-C considered a primary target for lipid-lowering therapy (11). However, large-scale studies have indicated a negative correlation between BL levels, particularly LDL-C, and the risk of intracerebral hemorrhage (ICH), certain cancers, and dementia (12–14), while exhibiting a protective effect against type 2 diabetes mellitus (T2DM) (15). These findings revealed inconsistencies in the association between BL levels and age-related disease risk.

Given the complex relationship between BL levels and age-related diseases, and the crucial role of TL in aging and age-related disease risk, the relationship between TL and BL has attracted widespread interest. Previous population-based prospective studies have consistently indicated a significant association between TL and TG, LDL-C, HDL-C and ApoA-1 (16–18). Cross-sectional (19) and cohort (20) studies have indicated that TL strongly correlates with ApoB. The relationship between the TL and BL, however, was found to be not significant in several observational studies (21–23). The existence of inconsistent outcomes introduces difficulties in making conclusive inferences about the causal relationship between TL and BL. However, the association between TL and BL observed in observational studies could be influenced by confounding variables, limited follow-up duration, small sample sizes and the potential for reverse causation (24). These factors may lead to misleading conclusions. Thus, the potential causality of TL in determining the BL level remains elusive, and vice versa.

Mendelian randomization (MR) is a more reliable method of causal inference that overcomes the limitations of observational studies (25, 26). MR uses genetic variation strongly associated with exposure factors as a tool, which can effectively avoid the effects of confounding factors and reverse causation (25, 27–29). With the identification of numerous variants associated with complex exposures through genome-wide association studies (GWAS), MR has gained widespread applicability (30, 31). In this study, we applied a two-sample bidirectional MR analysis to investigate the potential causal relationship between BL and TL. Given the intimate association between BL, TL and obesity (32), we performed a two-step mediation analysis to investigate the mediating pathway from BL to TL via obesity-related phenotypes, and vice versa.

## 2 Materials and methods

### 2.1 Study design

**Figure 1:**
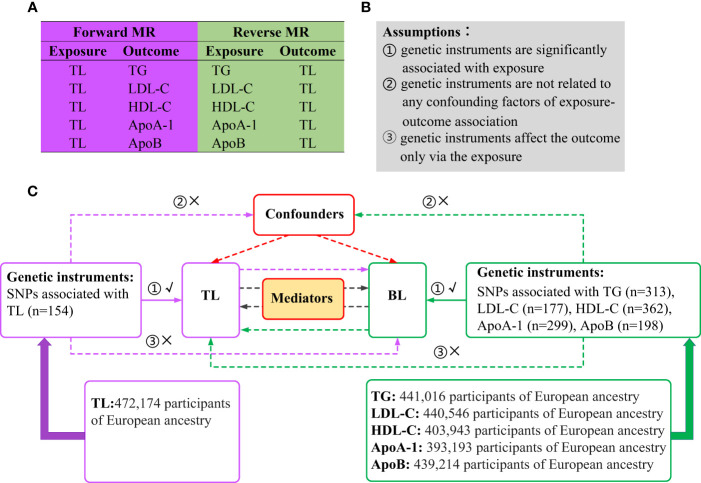
Overview of the study design. **(A)** Ten MR analyses investigating the bidirectional association between TL and BL. **(B)** MR analysis is underpinned by three core assumptions. **(C)** Outline of the study design. MR, Mendelian randomization; TL, telomere length; BL, blood lipids; TG, triglycerides; LDL-C, low-density lipoprotein cholesterol; HDL-C, high-density lipoprotein cholesterol; ApoA-1, apolipoprotein A-1; ApoB, apolipoprotein B; SNPs, single nucleotide polymorphisms.

A brief illustration of the bidirectional MR design is shown in **Figure 1**. BL is characterized by five generally assessed lipid traits. We assessed the causal relationship between TL and BL using forward-direction MR analysis. To ensure a comprehensive analysis, we adopted summary-level statistics from the most extensive GWAS conducted on TL. In the reverse MR analysis, we evaluated the correlation between the genetically predicted BL and TL. Summary-level statistics from the most comprehensive GWAS were also extracted for TG, LDL-C, HDL-C, ApoA-1 and ApoB. Therefore, we conducted 10 MR analyses to explore the bidirectional association between the TL and BL. The associations of BL on TL were adjusted via multivariable MR to eliminate potential pleiotropy (33). We also investigated mediation effects for TL on BL via mediation analysis and vice versa. MR analysis is underpinned by three core assumptions (**Figure 1**): genetic instruments are significantly associated with exposure; genetic instruments are unrelated to any confounding factors of the exposure-outcome association; and genetic instruments affect the outcome only via exposure (34). The analyses were restricted to individuals of European ancestry to minimize potential racial mismatches. Genotypes in the GWASs were imputed using the 1000 Genomes Project reference panels (35). An additive genetic model was used as the basis for analysis for all the GWAS summary statistics utilized in this study.

### 2.2 Data source and instruments

#### 2.2.1 Selection of genetic instruments

The genetic instruments were selected according to the three main assumptions of MR. Firstly, we filtered single-nucleotide polymorphisms (SNPs) at the genome-wide significance threshold (P < 5 × 10^−8^). To ensure independence among the genetic instruments, we also utilized linkage disequilibrium clumping (36) with r^2^ > 0.001 (clumping window of 10,000 kb). To meet the assumption that genetic instruments affect the outcome only through the exposure, we excluded outcome-related (P < 1×10^−5^) SNPs. PhenoScanner V2 (http://www.phenoscanner.medschl.cam.ac.uk) and GWAS Catalog (http://www.ebi.ac.uk/gwas) were used for investigating genetic associations with various phenotypes and traits. With the help of the online tools, we queried and removed SNPs significantly (P < 5 × 10^−8^) associated with potential confounders (37). In addition, incompatible and palindromic SNPs were removed when harmonizing the effect allele of each SNP between the summary statistics of exposure and outcome. Furthermore, we applied Steiger filtering to remove SNPs that may have a reverse potential causal direction, avoiding the association between each SNP and the outcome being stronger than that of the exposure (38).

#### 2.2.2 Data source and SNP selection for TL

Summary-level data for TL (n = 472,174) were obtained from the largest published GWAS in the UK Biobank (39). Using genome-wide significance filtering and linkage disequilibrium clumping, 154 independent SNPs significantly associated with TL were retained (**Supplementary Table 1**; **Figure 1**). After removing SNPs associated with potential confounders [i.e., diabetes, hypertension, smoking, and body mass index (BMI)] (40–43), 137 SNPs remained (**Supplementary Table 7**). For example, the index SNP at KIAA1429, which catalyzes the m6A methylation modification of RNA (44), was removed because of its significant association with BMI (rs1023767, P = 3.24 × 10–^8^). After the coding alleles in the summary statistics of TL were aligned with those of the outcome measures, 135, 135, 124, 128, and 135 SNPs remained to assess the associations between TL and TG, LDL-C, HDL-C, ApoA-1, and ApoB, respectively. None of these genetic instruments was removed by Steiger filtering, which explains the correctness of the causal direction for a single SNP. Detailed information on the number of SNPs preserved after each selection step is provided in **Supplementary Table 15**.

#### 2.2.3 Data source and SNP selection for BL

Summary statistics for TG (n = 441,016), LDL-C (n = 440,546), HDL-C (n = 403,943), Apo A-1 (n = 393,193), and ApoB (n = 439,214) were available from a comprehensive GWAS dataset from the UK Biobank (45). The mean (standard deviation [SD]) lipid concentrations were LDL-C 3.57 (0.87) mmol/L and HDL-C 1.45 (0.38) mmol/L, and the median TG was 1.50 (interquartile range = 1.11) mmol/L. The mean (SD) values for ApoB and ApoA-1 were 1.03 (0.24) g/L and 1.54 (0.27) g/L, respectively. Genome-wide significant filtering and linkage disequilibrium clumping identified 313 SNPs when TG was used as the exposure (**Supplementary Table 2**). After removing SNPs associated with the potential confounders (that is, diabetes, hypertension and smoking, and BMI) (46–49), 254 remained (**Supplementary Table 7**). By matching the TG and TL coding alleles, 236 SNPs were identified. Steiger filtering removed no SNPs, resulting in 236 genetic instruments being selected for LDL-C. Using the same selection procedures, 147, 303, 242, and 170 genetic instruments were selected for TG, HDL-C, ApoA-1 and ApoB, respectively (**Supplementary Tables 3–7,15**).

#### 2.2.4 Data source and SNP selection for potential mediators

Summary-level data of potential mediators (obesity-related phenotypes) were derived from comprehensive GWAS datasets, with BMI (n = 359,983), waist-to-hip ratio (WHR) (n = 224,459), hip circumference (HC) (n = 225,487) and waist circumference (WC) (n = 245,746) derived from the Genetic Investigation of Anthropometric Traits Consortium (50, 51). GWAS dataset for body fat percentage (BFP) (n = 331, 117) was derived from Neale Lab (http://www.nealelab.is). To ensure genome-wide significance and avoid potential confounding (GWAS Catalog was used to investigate each SNP to assess its associations with confounding factors), 69, 257, 31, 75 and 65 genetic instruments were eventually selected for BMI, BFP, WHR, HC and WC (**Supplementary Tables 8–12**).

### 2.3 Statistical analysis

#### 2.3.1 Univariable MR

R^2^ was calculated to estimate the proportion of variance in liability explained by genetic instruments (52). The F-statistic was calculated to validate the strength of the association between genetic instruments and exposure, and a threshold of F-statistic > 10 was suggested for MR analysis (53). The inverse variance weighted (IVW) method was adopted as the principal MR analytical approach to assess potential associations between TL and BL (54). In addition, we utilized the Mendelian Randomization-Egger (MR-Egger) (55), weighted median (56), and weighted mode (57) as alternative analysis methods. Bonferroni-corrected P < 0.005 (0.05/10 = 0.005) was used to determine statistical significance in the univariable MR analysis, and beta (β) with 95% confidence intervals (CI) were applied to estimate the degree of causal relationships. We then evaluated the heterogeneity for the IVW and MR-Egger methods using Cochran’s Q statistics (58), where P < 0.05 suggesting apparent heterogeneity. The IVW random effects (IVW-RE) method was used for heterogeneous SNPs. We also performed tests for horizontal pleiotropy using the MR-Egger regression intercept, and statistical significance was set at P < 0.05. Additionally, we applied the MR pleiotropy residual sum and outlier (MR-PRESSO) test to identify and eliminate horizontal pleiotropic outliers (59).

#### 2.3.2 Multivariable MR

The special BL traits share correlation in terms of function and composition, and there were SNPs associated with at least two of the five BL traits. Considering these relationships, we performed multivariable MR (60) analysis to simultaneously estimate the causal effect of each BL trait on TL conditioned on related BL traits. We designed two models to correct for both measured and unmeasured pleiotropy, using the multivariable MR extension of the IVW and MR-Egger method. Model 1 included TG, LDL-C and ApoB, as TG combines with ApoB to form very-low-density lipoprotein cholesterol (VLDL-C) particles, and these VLDL-C particles transport TG in the bloodstream to other tissues, gradually converting into LDL-C (61). Model 2 included HDL-C and ApoA-1, as ApoA-1 is the major structural protein of HDL-C. p < 0.05 was considered significant in the multivariate MR analysis.

#### 2.3.3 Mediation analysis

For significant MR associations, two-step MR analysis was applied to evaluate mediating effects. In the first step, genetic instruments for exposure were used to access the causal effect of the exposure on the potential mediators. In the second step, genetic instruments for the identified mediators were used to estimate the causal effect of the potential mediators on outcome. When there was evidence that TL influenced the mediator, which in turn influenced BL, we utilized the “product of coefficients” method (62) to assess the mediation effect of TL on BL via each potential mediator in the forward-direction MR analysis, and vice versa. Standard errors for the indirect effects were derived by using the delta method (63). Given that obesity-related phenotypes are correlated with both BL and TL (64, 65), it is plausible that obesity-related measurements could act as mediators between TL and BL. A p-value < 0.05 suggests statistical significance in mediation analysis.

All above MR analysis were performed using the TwoSampleMR, MRPRESSO and MendelianRandomization packages in R (version 4.2.0; www.r-project.org/).

## 3 Results

### 3.1 Estimates of the causal effect of TL on BL

#### 3.1.1 Univariable MR

**Figure 2:**
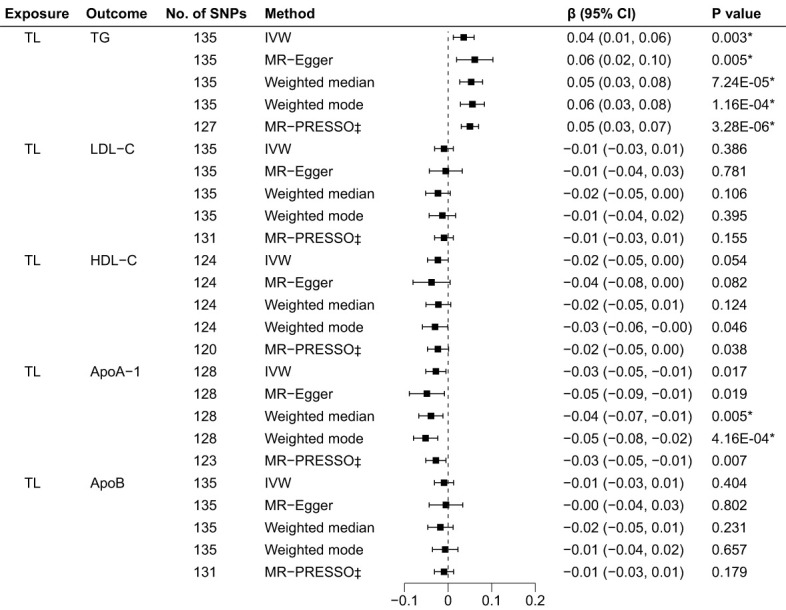
Associations of TL and BL in the forward MR analyses. TL, telomere length; BL, blood lipids; MR, Mendelian randomization; SNPs, single nucleotide polymorphisms; OR, odds ratio; CI, confidence interval; IVW, inverse variance weighted (random-effects model); TG, triglycerides; LDL-C, low-density lipoprotein cholesterol; HDL-C, high-density lipoprotein cholesterol; ApoA-1, apolipoprotein A-1; ApoB, apolipoprotein B; *Bonferroni-corrected P < 0.005 (0.05/10 = 0.005) was used to determine statistical significance; ‡MRgnifica instrumental variable outlier removed.

The R^2^ and F-statistics indicated that all SNPs were sufficiently powerful to predict the exposure of interest (**Supplementary Table 13**). The results of the forward MR analyses are shown in **Figure 2**, and the scatter and forest plots are presented in **Supplementary Figures 1, 2**. The TL were significantly positive associated with the TG levels. The estimates of causal effect for each SD longer TL were 0.04 SD higher level of TG (95% CI: 0.01–0.06; p = 0.003) in the IVW analysis. No significant correlation was found for LDL-C (β = -0.01, 95% CI: -0.03 to 0.01, p = 0.386), HDL-C (β = -0.02, 95% CI: -0.05 to 3.64E-04, p = 0.054) and ApoB (β = -0.01, 95% CI: -0.03 to 0.01, p = 0.404) after Bonferroni correction. Using other MR methods, i.e., MR-Egger, weighted median and weighted mode—all estimates of the causal effects were consistent with those of IVW. Additionally, although IVW (β = -0.03, 95% CI: -0.05 to -0.01, p = 0.017) and MR-Egger (β = -0.05, 95% CI: -0.09 to -0.01, p = 0.019) methods showed on significant association between TL on ApoA-1 after Bonferroni correction, weighted median (β = -0.04, 95% CI: -0.07 to -0.01, p = 0.005) and weighted mode (β = -0.05, 95% CI: -0.08 to -0.01 = 2, p = 4.16E-04) methods indicated significant causal effects for TL on ApoA-1, suggesting implicit association between TL and ApoA-1. The MR-PRESSO test identified 8 outlier SNPs for TG, 4 outlier SNPs for LDL-C, 4 for HDL-C, 5 for ApoA-1 and 4 for ApoB, respectively (**Supplementary Table 14**). After removing outlier SNPs which had horizontal pleiotropy, the corrected MR-PRESSO analysis showed consistent causal estimates with IVW analysis.

Cochran’s Q test indicated different degrees of heterogeneity (**Table 1**; all P-values of Cochran’s Q < 0.05), whereas funnel plots revealed no perceptible heterogeneity (**Supplementary Figure 3**). Furthermore, the leave-one-out analysis showed no significant changes after eliminating any single SNP, suggesting the stability of the observed associations (**Supplementary Figure 4**).

**Table 1:** Heterogeneity and horizontal pleiotropy analysis.

Exposure	Outcome	MR‐Egger regression		Heterogeneity test (IVW)		Heterogeneity test (MR Egger)	
		Intercept	p_intercept	Q	Q_pval	Q	Q_pval
TL	TG	-8.77E-04	0.148	331.655	9.14E-19	326.469	2.77E-18
TL	LDL-C	-1.43E-04	0.935	242.583	2.84E-08	242.460	2.14E-08
TL	HDL-C	4.92E-04	0.420	293.220	6.26E-16	291.655	6.38E-16
TL	ApoA-1	7.27E-04	0.224	296.231	1.49E-15	292.756	2.67E-15
TL	ApoB	-1.51E-04	0.787	257.114	8.68E-10	256.976	6.40E-10
TG	TL	-8.77E-04	0.148	354.131	7.91E-07	349.514	1.43E-06
LDL-C	TL	-1.43E-04	0.935	247.071	3.47E-07	247.054	2.64E-07
HDL-C	TL	4.92E-04	0.420	575.015	3.12E-19	574.996	2.26E-19
ApoA-1	TL	7.27E-04	0.224	439.836	8.73E-14	437.771	1.03E-13
ApoB	TL	-1.51E-04	0.787	283.153	8.74E-08	281.066	1.03E-07

TL, telomere length; BL, blood lipids; IVW, inverse variance weighted; TG, triglycerides; LDL-C, low-density lipoprotein cholesterol; HDL-C, high-density lipoprotein cholesterol; ApoA-1, apolipoprotein A-1; ApoB, apolipoprotein B.

#### 3.1.2 Mediation analysis

We conducted two-step MR analysis to investigate the mediating pathway from TL to TG via five obesity-related phenotypes, including BMI, BFP, WHR, HC and WC. In the first step, we estimated the correlation across TL and potential mediators. Among the five obesity-related phenotypes, we found positive significant association between TL and WC (β = 0.07, 95% CI: 7.64E-04 to 0.13, p = 0.047) (**Supplementary Table 16**). In the second step, we evaluated the causal effects for obesity-related phenotypes on TG, and we only identified positive causal effect of WC (β=0.09, 95% CI: 0.04 to 0.13, p = 2.23E-04) on TG. Finally, we assessed the mediation effect of TL on TG acting through WC, and no significant mediation effect was found (β = 0.01, 95% CI: -8.91E-04 to 0.01, p = 0.081) (**Table 2**).

**Table 2:** The mediation effect of TL on TG via obesity-related phenotypes.

Exposure	Outcome	Mediator	Total effect	Direct effect A	Direct effect B	Mediation effect		Mediated proportion (%) (95% CI)
			*β* (95% CI)	*β* (95% CI)	*β* (95% CI)	*β* (95% CI)	P	
TL	TG	BMI	0.03 (0.01, 0.06)	-1.31E-04 (-0.05, 0.05)	0.11 (0.07, 0.15)	-1.42E-05 (-0.01, 0.01)	0.996	-0.04 (-18.39, 18.30)
		BFP	0.03 (0.01, 0.06)	-0.02 (-0.04, 5.35E-04)	0.23 (0.19, 0.27)	-4.97E-03 (-0.01, 2.07E-04)	0.059	-15.49 (-31.62, 0.64)
		WHR	0.02 (3.45 E-03, 0.03)	0.03 (-0.04, 0.09)	0.11 (0.01, 0.20)	-1.93E-03 (-0.01, 2.84 E-03)	0.411	-12.09 (-42.02, 17.84)
		HC	0.03 (0.01, 0.06)	0.03 (-0.04, 0.10)	0.01 (-0.02, 0.05)	4.42E-04 (-1.55E-03, 2.43E-03)	0.565	1.38 (-4.82, 7.58)
		WC	0.03 (0.01, 0.06)	0.07 (7.64E-04, 0.13)	0.09 (0.04, 0.13)	0.01 (-8.91E-04, 0.01)	0.081	18.09 (-2.78, 38.96)

TL, telomere length; TG, triglycerides; BMI, body mass index; BFP, body fat percentage; WHR, waist-to-hip ratio; HC, hip circumference; WC, waist circumference. Total effect: the effect of TL on TG; Direct effect: the effect of TL on TG, not explained by the mediator; Mediation effect: the effect of TL on TG acting through the mediator.

### 3.2 Estimates of the causal effect of BL on TL

#### 3.2.1 Univariable MR

**Figure 3:**
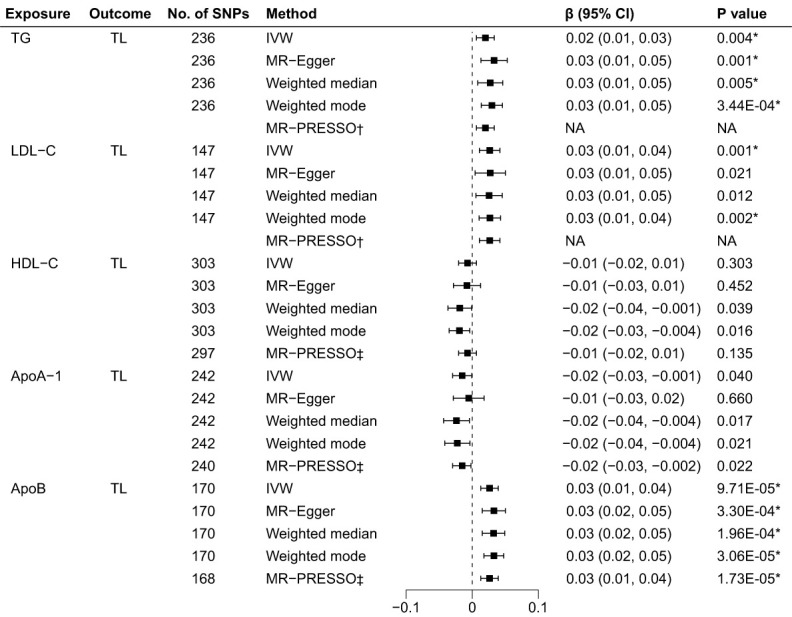
Associations of BL and TL in the reverse MR analyses. BL, blood lipids; TL, telomere length; MR, Mendelian randomization; SNPs, single nucleotide polymorphisms; IVW, inverse variance weighted; OR, odds ratio; CI, confidence interval; TG, triglycerides; LDL-C, low-density lipoprotein cholesterol; HDL-C, high-density lipoprotein cholesterol; ApoA-1, apolipoprotein A-1; ApoB, apolipoprotein B; *Bonferroni-corrected P < 0.005 (0.05/10 = 0.005) was used to determine statistical significance; †No outlier detected; ‡MRtected; instrumental variable outlier removed.

As illustrated in **Figure 3**, the genetically predicted TG, LDL-C, and ApoB levels were positively correlated to TL after Bonferroni correction. Using IVW in the reverse MR analyses, the estimates of causal effect for each SD higher level of TG was 0.02 SD longer TL (95% CI: 0.01 to 0.03, p = 0.004). Similar estimates of causal effect were observed for LDL-C (β = 0.03, 95% CI: 0.01 to 0.04, p = 0.001) and ApoB (β = 0.03, 95% CI: 0.01 to 0.04, p = 9.71×10–^5^) on TL. In contrast, the estimates of causal effect were nonsignificant across both HDL-C (β = -0.01, 95%CI: -0.02 to 0.006, p = 0.303) and ApoA-1 (β = -0.02, 95%CI: -0.03 to -0.001, p = 0.040) on TL. Other MR approach also suggested similar results. Furthermore, the causal effects for LDL-C on TL estimated through MR-Egger (β = 0.03, 95% CI: 0.01 to 0.05, p = 0.021) and weighted median (β = 0.03, 95% CI: 0.01 to 0.05, p = 0.012) did not achieve significance threshold after Bonferroni correction, indicating a proposing correlation. The MR-PRESSO test identified 6 outlier SNPs for HDL-C, 2 for ApoA-1 and 2 for ApoB. No outlier SNPs were identified for the LDL-C or TG levels (**Supplementary Table 14**). The MR-PRESSO analysis exhibited compatible results with the IVW analysis. The scatter plots (**Supplementary Figure 5**) and forest plots (**Supplementary Figure 6**) show consistent estimates of the causal effect of BL on TL.

The MR-Egger regression test showed no evidence of horizontal pleiotropy and Cochran’s Q test indicated evident heterogeneity (**Table 1**). However, funnel plots (**Supplementary Figure 7**) suggested no potential existence of heterogeneity. The leave-one-out analysis also revealed the robustness of the observed results (**Supplementary Figure 8**).

#### 3.2.2 Multivariable MR

Considering the correlation across BL traits, we performed multivariable MR analysis to simultaneously estimate the direct effect of special BL trait on TL conditioned on other BL traits (**Table 3**). The independent instruments used for multivariable MR are listed in **Supplementary Table 14**. The multivariable IVW estimates for TG (β = 0.04, 95% CI: -0.06 to 0.14, p = 0.437), LDL-C (β = -0.20, 95% CI: -0.95 to 0.55, p = 0.598) and ApoB (β = 0.18, 95% CI: -0.46 to 0.81, p = 0.586) on TL were not significant, which was inconsistent with univariable IVW estimates. The results indicated potentially unstable relationship between TG, LDL-C, ApoB and TL. The multivariable IVW estimates for HDL-C (β = 0.002, 95% CI: -0.11 to 0.11, p = 0.966) and ApoA-1 (β = -0.02, 95% CI: -0.13 to 0.09, p = 0.714) on TL were also not significant.

**Table 3:** The multivariable effect of BL on TL.

Model	Exposure	No. of SNPs	Method	β (95% CI)	P
Model1	TG	5	IVW	0.04 (-0.06, 0.14)	0.437
			MR-Egger	0.06 (-0.18, 0.29)	0.639
	LDL-C	5	IVW	-0.20 (-0.95, 0.55)	0.598
			MR-Egger	0.16 (-1.31,1.62)	0.835
	ApoB	5	IVW	0.18 (-0.46, 0.81)	0.586
			MR-Egger	-0.08 (-1.07,0.90)	0.867
Model2	HDL-C	76	IVW	0.002 (-0.11, 0.11)	0.966
			MR-Egger	5.12E-04 (-0.05, 0.05)	0.983
	ApoA-1	76	IVW	-0.02 (-0.13, 0.09)	0.71
			MR-Egger	-2.58E-03 (-0.05, 0.05)	0.920

TG, triglycerides; LDL-C, low-density lipoprotein cholesterol; ApoB, apolipoprotein B; HDL-C, high-density lipoprotein cholesterol; ApoA-1, apolipoprotein A-1; IVW, inverse variance weighted.

#### 3.2.3 Mediation analysis

We conducted two-step MR analysis to explore whether the effect of TG, LDL-C and ApoB on TL was mediated via obesity-related phenotypes, i.e., BMI, BFP, WHR, HC and WC. In the first step, we assessed the causal effects for TG, LDL-C and ApoB on the five obesity-related phenotypes. Significant associations were identified for TG on WHR (β = 0.09, 95% CI: 0.04 to 0.14, p = 5.61E-04) and HC (β = -0.09, 95% CI: -0.14 to -0.04, p = 2.00E-04) (**Supplementary Table 17**). The results also showed significant causal effects for LDL-C on BMI (β = -0.08, 95% CI: -0.12 to -0.04, p = 1.57E-04) and WHR (β = -0.06, 95% CI: -0.10 to -0.02; p = 0.006), as well as AopB on HC (β = -0.07, 95% CI: -0.13 to -0.01, p = 0.016). In the second step, we estimated the causal effects for BMI, WHR and HC on TL, and we found evidence that BMI (β = -0.04, 95% CI: -0.07 to -0.01, p = 0.011) was significantly associated with TL. Ultimately, we assessed the mediation effects for TG, LDL-C and ApoB on TL via potential mediators. We only identified significant mediation effect of LDL-C on TL acting via BMI (β = 2.97E-03, 95% CI: 1.44E-04 to 5.79E-03, p = 0.035) with a mediated proportion of 12.83% (95% CI: 0.62% to 25.04%) (**Table 4**).

**Table 4:** The mediation effect of TG, LDL-C and ApoB on TL via obesity-related phenotypes.

Exposure	Outcome	Mediator	Total effect	Direct effect A	Direct effect B	Mediation effect		Mediated proportion (%) (95% CI)
			*β* (95% CI)	*β* (95% CI)	*β* (95% CI)	*β* (95% CI)	P	
TG	TL	BMI	0.02 (3.45E-03, 0.03)	-0.01 (-0.05, 0.02)	-0.04 (-0.07, -0.01)	3.91E-04 (-1.02E-03, 1.80E-03)	0.560	2.46 (-6.39, 11.30)
		BFP	0.02 (3.45E-03, 0.03)	-3.75E-03 (-0.02, 0.02)	-0.06 (-0.09, -0.04)	2.40E-04 (-1.14E-03, 1.62E-03)	0.729	1.51 (-7.19, 10.20)
		WHR	0.02 (3.45E-03, 0.03)	0.09 (0.04, 0.14)	-0.02 (-0.07, 0.03)	-1.92E-03 (-0.01, 2.84E-03)	0.411	-12.09 (-42.02, 17.84)
		HC	0.02 (3.45E-03, 0.03)	-0.09 (-0.14, -0.04)	0.02 (-4.85E-03, 0.04)	-1.78E-03 (-4.28E-03, 7.19E-04)	0.150	-11.19 (-26.90, 4.52)
		WC	0.02 (3.45E-03, 0.03)	0.05 (-1.77E-03, 0.10)	0.01 (-0.02, 0.05)	5.10E-04 (-1.42E-03, 2.45E-03)	0.564	3.20 (-8.97, 15.38)
LDL-C	TL	BMI	0.02 (0.01, 0.04)	-0.08 (-0.12, -0.04)	-0.04 (-0.07, -0.01)	2.97E-03 (1.44E-04, 5.79E-03)	0.035	12.83 (0.62, 25.04)
		BFP	0.02 (0.01, 0.04)	-0.01(-0.03, 3.83E-03)	-0.06 (-0.09, -0.04)	9.48E-04 (-3.31E-04, 2.22E-03)	0.139	4.10 (-1.43, 9.63)
		WHR	0.02 (0.01, 0.04)	-0.06 (-0.10, -0.02)	-0.02 (-0.07, 0.03)	1.24E-03 (1.95E-03, 4.43E-03)	0.418	5.38 (-8.41, 19.17)
		HC	0.02 (0.01, 0.04)	-0.04 (-0.09, 0.01)	0.02 (-4.85E-03, 0.04)	-7.43E-04 (-2.25E-03, 7.68E-04)	0.287	-3.21 (-9.75, 3.32)
		WC	0.02 (0.01, 0.04)	-0.04 (-0.09, 4.65E-03)	0.01 (-0.02, 0.05)	4.68E-04 (-2.28E-03, 1.34E-03)	0.566	-2.02 (-9.86, 5.81)
ApoB	TL	BMI	0.02 (0.01, 0.04)	-0.02 (-0.07, 0.03)	-0.04 (-0.07, -0.01)	7.60E-04 (-1.25E-03, 2.78E-03)	0.431	3.12 (-5.17, 11.41)
		BFP	0.02 (0.01, 0.04)	-0.01 (-0.03, 4.19E-03)	-0.06 (-0.09, -0.04)	7.62E-04 (-3.35E-04, 1.86E-03)	0.165	3.13 (-1.38, 7.63)
		WHR	0.02 (0.01, 0.04)	2.09E-03 (-0.05, 0.05)	-0.02 (-0.07, 0.03)	-4.57 E-05 (-1.64E-03, 1.55E-03)	0.931	-0.19 (-6.75, 6.37)
		HC	0.02 (0.01, 0.04)	-0.07 (-0.13, -0.01)	0.02 (-4.85E-03, 0.04)	-1.33E-03 (-3.46E-03, 7.83E-04)	0.191	-5.49 (-1.42, 3.22)
		WC	0.02 (0.01, 0.04)	-0.01 (-0.07, 0.04)	0.01 (-0.02, 0.05)	1.45E-04 (-1.42E-03, 1.13E-03)	0.713	-0.60 (-5.84, 4.64)

TG, triglycerides; LDL-C, low-density lipoprotein cholesterol; ApoB, apolipoprotein B; TL, telomere length; BMI, body mass index; BFP, body fat percentage; WHR, waist-to-hip ratio; HC, hip circumference; WC, waist circumference. Total effect: the effect of TL on TG; Direct effect: the effect of TL on TG, not explained by the mediator; Mediation effect: the effect of TL on TG acting through the mediator.

## 4 Discussion

Previous research has reported inconsistent associations between TL and lipid traits (66–71), which poses challenges in drawing conclusive inferences about their causal relationship. In this study, we attempted to disentangle the causal relationship between TL and BL by leveraging substantial sample sizes and GWAS summary statistics. Given that TL serves as a clinical indicator of aging and age-related disease risk, clarifying this association is of great significance, because BL levels are also associated with these diseases (4, 5).

Our MR analyses provided reliable and robust findings regarding the associations between TL and BL, revealing the causal effect of TL on specific BL, and vice versa. Specifically, in the forward MR analyses, TL was significantly positively associated with TG levels, indicating that a longer TL predicted higher TG levels. In the reverse MR analysis, genetically predicted TG, LDL-C, and ApoB levels were positively correlated with TL, suggesting that higher TG, LDL-C, and ApoB levels predicted longer TL, although this relationship was not observed in the multivariate MR analysis. Additionally, we conducted mediation analysis to estimate potential mediating factors; analysis of TL with TG showed no significant mediation effects acting through obesity-related phenotypes, while the impact of LDL-C on TL was partially mediated by BMI, although the indirect effect was smaller than the total effect.

Our study found that higher TG, LDL-C, and ApoB levels predicted longer TL. Although our MR findings conflict with several relatively smaller observational studies (67, 72), they align with the results of the largest cross-sectional study conducted to date investigating these relationships (68). This study reported that higher levels of TG, LDL-C, and ApoB were associated with 0.48,1.04, and 0.96 years of age‐related TL change, respectively. This potentially beneficial role of BL in TL may facilitate prevention strategies and interventions directed toward clinical aging and age-related diseases. Multiple studies have contributed to understanding this relationship. A large-scale study of over 500,000 Chinese adults found that lower plasma LDL-C and TG levels were associated with increased ICH risk (12). The underlying mechanism is not fully understood but may be related to the increased vascular wall permeability associated with lower cholesterol levels (73). Additionally, higher LDL-C levels have shown an inverse correlation with dementia, indicating a potential protective role against cognitive decline (14). MR analysis also supported the association between lower LDL-C levels and increased risk of ICH and dementia, raising concerns about excessively low LDL-C levels (74). Moreover, LDL-C has been found to have a protective effect against type 2 diabetes (T2DM) (75), which may explain the slight increase in T2DM risk associated with statin therapy (15). The association between LDL-C levels and cancer is also inconsistent with its relationship to cardiovascular disease, with some studies showing a positive association between low LDL-C levels and cancer risk (13). These observations have implications in understanding the potentially beneficial effects of BL on telomere-related aging and age-related diseases. It is crucial to consider the delicate balance among cardiovascular benefits, potential aging, and age-related disease risks when managing LDL-C levels. Further research is necessary to elucidate the underlying mechanisms and develop targeted interventions to optimize health outcomes in individuals at risk for age-related conditions.

However, the mechanisms underlying the association between the TL and BL remain unclear. Oxidative and chronic inflammatory stress are thought to play crucial roles (76). The occurrence of dyslipidemia is frequently accompanied by changes in some inflammatory markers (77). It is worth noting that TL has been shown to be correlated with levels of inflammatory markers (67, 69). Oxidative stress is considered a major driving factor of telomere attrition (78, 79). Furthermore, oxidative stress directly affects lipid metabolism, leading to abnormal lipid levels. TL serves as a marker of DNA damage, and telomere dysfunction is caused by critically short telomeres or structural changes, ultimately resulting in replicative cell senescence and chromosomal instability, both of which are hallmarks of aging. However, studies have also found that longer telomeres are associated with a higher risk of incident myocardial infarction in healthy participants aged 65 years or older (18). A plausible explanation could be that telomeres inhibit further replication in senescent cells. Specifically, telomere attrition may lead to replicative senescence, which may serve as a mechanism for restricting elevated BL levels and atherosclerosis progression (80, 81), potentially similar to their inhibitory effects on carcinogenesis (82). Shortening of TL and cessation of proliferation in aging cell lines within the endothelium may be equally important as long telomeres in preventing the accumulation of DNA mutations. Overall, these findings partially explain the relationship between BL and TL. Nonetheless, future cellular and molecular research is necessary to elucidate these potential mechanisms.

We conducted a bidirectional two-step MR mediation analysis; the analysis of TL with TG showed no significant mediation effects, whereas the reverse MR results indicated that the protective effect of higher LDL-C levels on TL was partially mediated through a reduction in BMI, although the indirect effect was smaller than the total effect. In the first MR step, univariate MR established a causal relationship between LDL-C and BMI, showing that increased LDL-C levels were associated with decreased BMI. Previous studies reported inconsistent results regarding the association between BMI and BL. Several studies have reported a negative correlation between BMI and LDL-C levels (64, 83). Additionally, some studies have shown a nonlinear relationship between BMI and LDL-C (84), with LDL-C levels tending to plateau or decrease with increasing BMI in overweight populations (85). Furthermore, the association between BMI and LDL-C level may vary across sex and age subgroups. The diminishing correlation between BMI and LDL-C levels suggests metabolic impairment due to aging or other metabolic disorders. These findings are consistent with our first-step estimations. The second step of our MR analysis provided evidence that a genetically predicted lower BMI was associated with longer TL. Several published MR studies have reported causal evidence for BMI as a risk factor for TL or related phenotypes (86, 87), consistent with the estimations from the second step of our mediation analysis. Mechanistically, obesity-related metabolic dysregulation leads to oxidative stress, resulting in telomere shortening (88). Furthermore, obesity-induced inflammation partially mediates the negative association between BMI and TL (87, 89). In conclusion, these studies provided compelling evidence supporting the mediating effect of BMI.

Our study exhibits several strengths. First, we utilized summary statistics derived from large-scale GWASs to provide a solid foundation for our research. Furthermore, we employed a range of techniques to minimize the risk of violating the assumptions of MR, including the evaluation of index SNP associations with confounders, the use of Steiger filtering to effectively reduce the potential influence of reverse causation driven by genetic instruments and the selection of a primary method known for its resistance to pleiotropy, along with sensitivity analyses using alternative methods. If increasing blood lipid levels indeed provide protective effects with respect to telomeres, it could be a modifiable factor that could potentially help mitigate the risk of age-related diseases.

Our study also has several limitations that should be acknowledged. First, the MR approach relies on genetic instruments to represent lifelong differences in exposure levels, assuming that these instruments accurately estimate causal effects on the outcome. However, it is crucial to consider that developmental adaptation can alter the effects of these genetic instruments on outcomes (90). In addition, two-sample MR methods rely on GWAS summary statistics and assume a linear relationship between exposure and outcomes. Finally, as both the exposure and outcome in this study were derived from European populations, caution should be exercised when generalizing the research findings to other racial or ethnic groups.

In summary, this study employed large-scale exposure and outcome GWAS data for MR analysis to elucidate the causal relationship between TL and BL. We found robust genetic evidence supporting the causal effects of TL on TG, whereas reverse MR analyses identified protective causal relationships among TG, LDL-C, and ApoB on TL, with BMI partially mediating the causal effect of LDL-C on TL. Our findings provide insights into preventive strategies and interventions targeting TL-related aging and age-related diseases.

## Data availability statement

Publicly available datasets were analyzed in this study. All summary level GWAS results are publicly available from the IEUOpenGWAS platform, accessible at https://gwas.mrcieu.ac.uk/.

## Ethics statement

The current Mendelian randomization analysis utilized summary data from prior studies that had obtained written informed consent and ethical approval. The secondary analysis of summary data does not necessitate an additional ethical permit.

## Author contributions

SY: Conceptualization, Data curation, Formal analysis, Writing – original draft, Writing – review & editing. XW: Validation, Writing – review & editing. YL: Validation, Writing – review & editing. LZ: Validation, Writing – review & editing. GG: Project administration, Supervision, Writing – review & editing. MW: Project administration, Supervision, Validation, Funding acquisition, Writing – review & editing.
