# Supplementary material for: The association between telomere length and blood lipids: a bidirectional two-sample Mendelian randomization study
Source: Front Endocrinol (Lausanne). 2024 May 28;15:1338698. doi: 10.3389/fendo.2024.1338698 (PMC11165217; doi:10.3389/fendo.2024.1338698)
Supplement: Supplementary file 1 [file DataSheet_1.docx]

Supplementary Figures

**Supplementary Figure 1** Scatter plots of the association between TL and BL in the forward MR analyses. **(Page 2)**

**Supplementary Figure 2** The forest plots of the association between TL and BL in the forward MR analyses. **(Page 3)**

**Supplementary Figure 3** The funnel plots of the association between TL and BL in the forward MR analyses. **(Page 4)**

**Supplementary Figure 4** The leave-one-out analysis of the association between TL and BL in the forward MR analyses. **(Page 5)**

**Supplementary Figure 5** Scatter plots of the association between BL and TL in the reverse MR analyses. **(Page 6)**

**Supplementary Figure 6** The forest plots of the association between BL and TL in the reverse MR analyses. **(Page 7)**

**Supplementary Figure 7** The funnel plots of the association between BL and TL in the reverse MR analyses. **(Page 8)**

**Supplementary Figure 8** The leave-one-out analysis of the association between BL and TL in the reverse MR analyses. **(Page 9)**


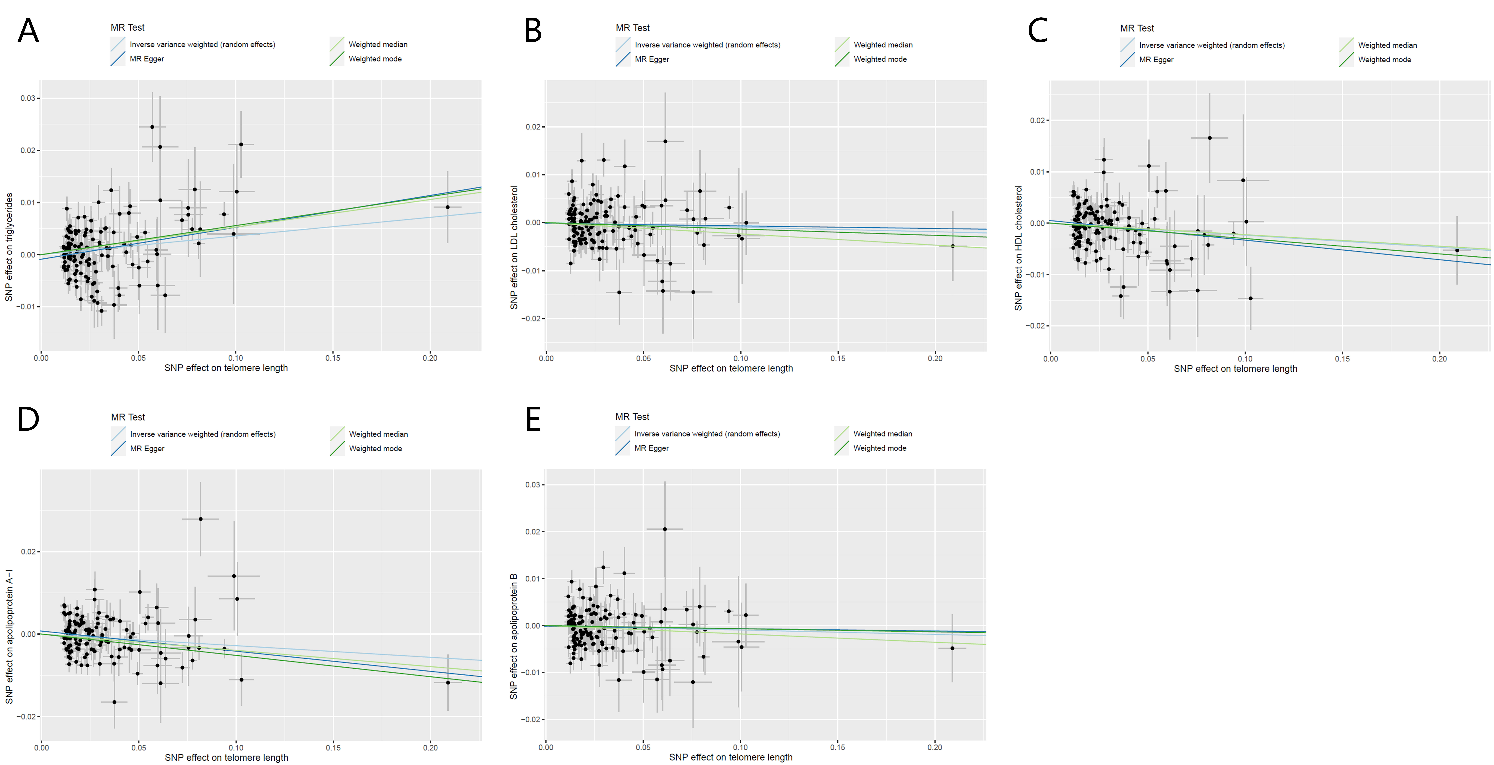


**Supplementary Figure 1.** Scatter plots of the association between TL and BL in the forward MR analyses. (A)TL-triglycerides (TG); (B)TL-low-density lipoprotein cholesterol (LDL-C); (C)TL-high-density lipoprotein cholesterol (HDL-C); (D)TL-apolipoprotein A-1 (ApoA-1); (E)TL- apolipoprotein B (ApoB). Each line's slope reflects the estimated association effect of TL on BL, as determined by distinct MR methods. TL, telomere length; BL, blood lipids; MR, Mendelian randomization.


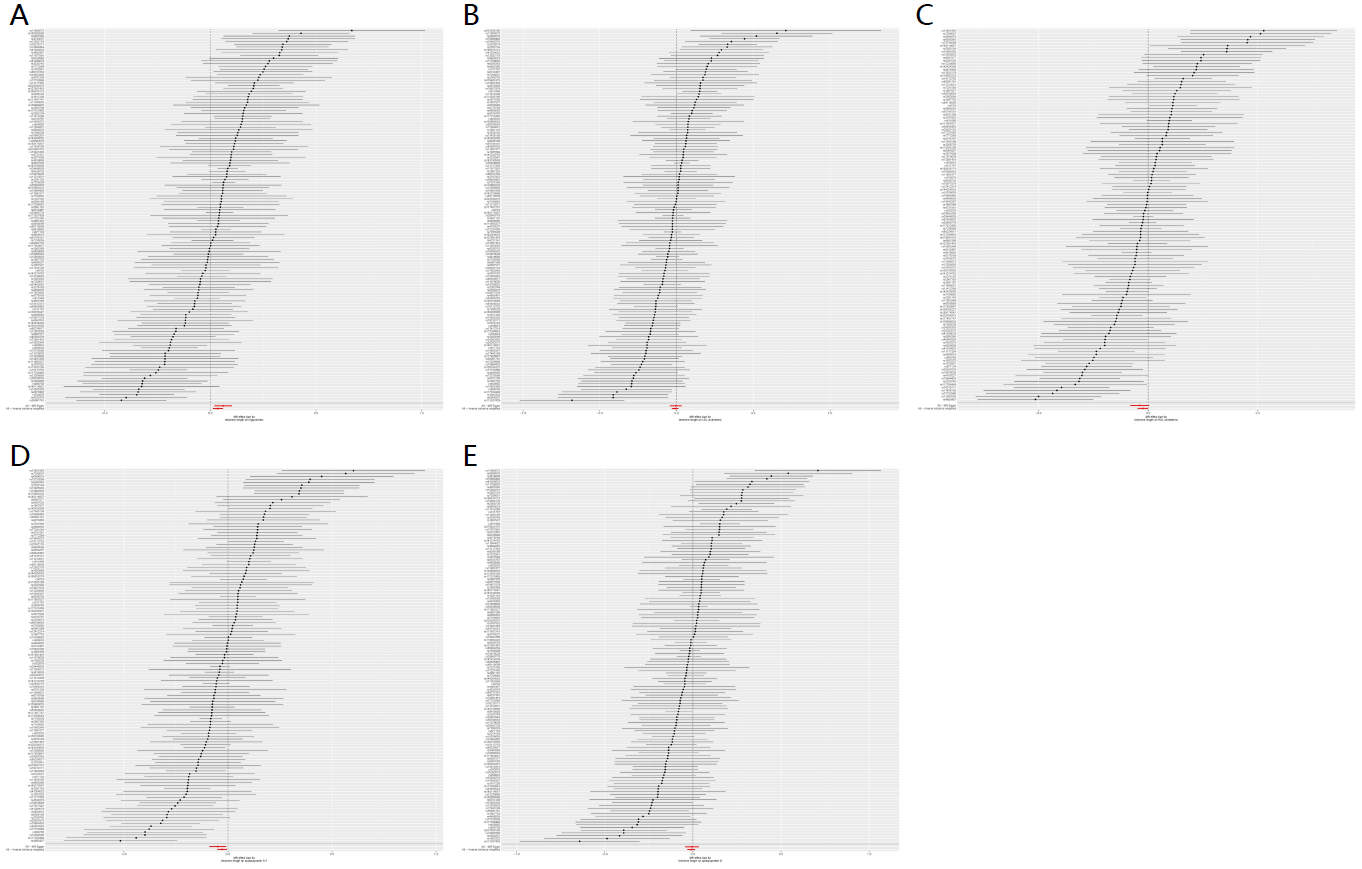


**Supplementary Figure 2.** The forest plots of the association between TL and BL in the forward MR analyses. (A)TL-triglycerides (TG); (B)TL-low-density lipoprotein cholesterol (LDL-C); (C)TL-high-density lipoprotein cholesterol (HDL-C); (D)TL-apolipoprotein A-1 (ApoA-1). (E)TL- apolipoprotein B (ApoB). TL, telomere length; BL, blood lipids.


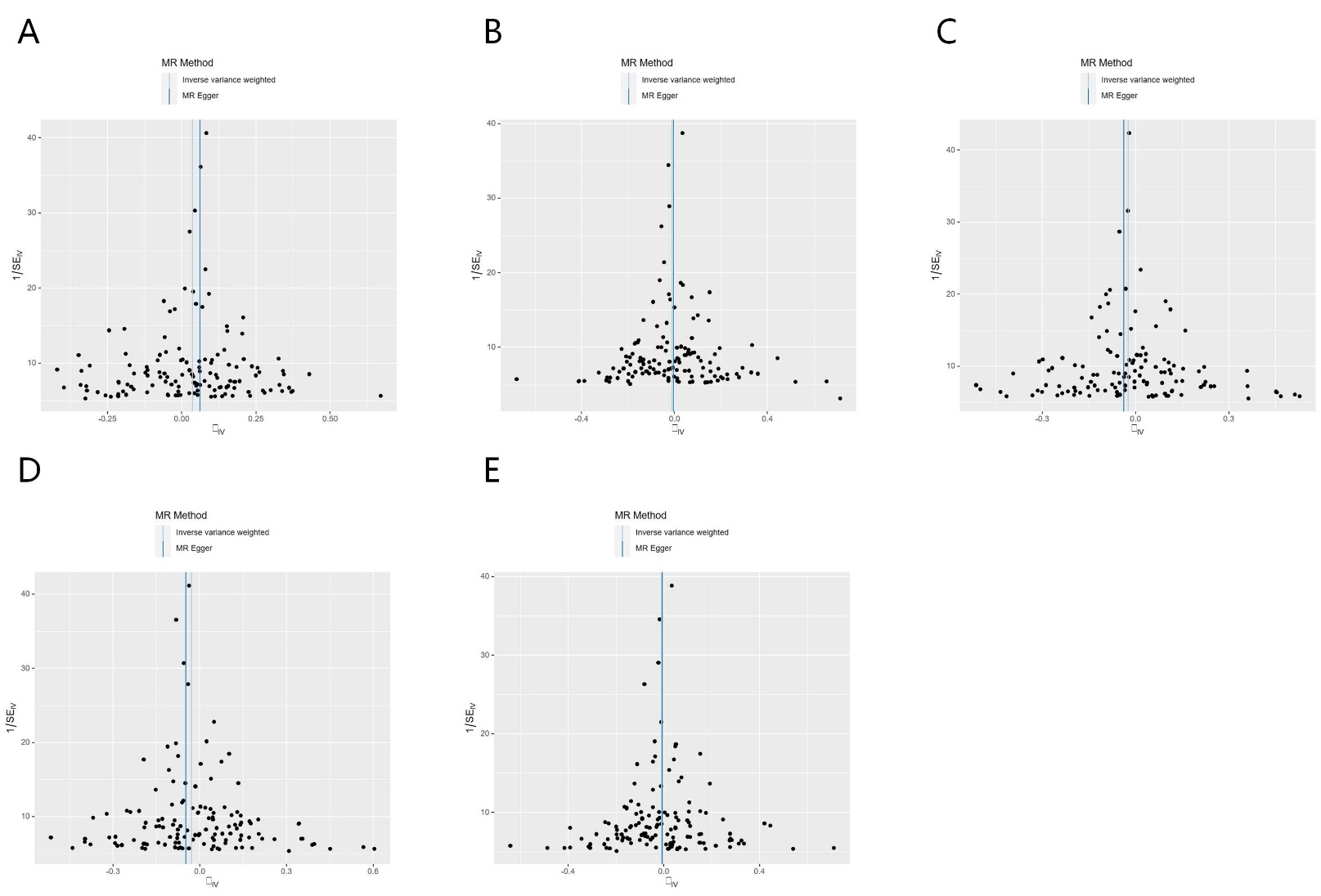


**Supplementary Figure 3.** The funnel plots of the association between TL and BL in the forward MR analyses. (A)TL-triglycerides (TG); (B)TL-low-density lipoprotein cholesterol (LDL-C); (C)TL-high-density lipoprotein cholesterol (HDL-C); (D)TL-apolipoprotein A-1 (ApoA-1). (E)TL- apolipoprotein B (ApoB). TL, telomere length; BL, blood lipids.


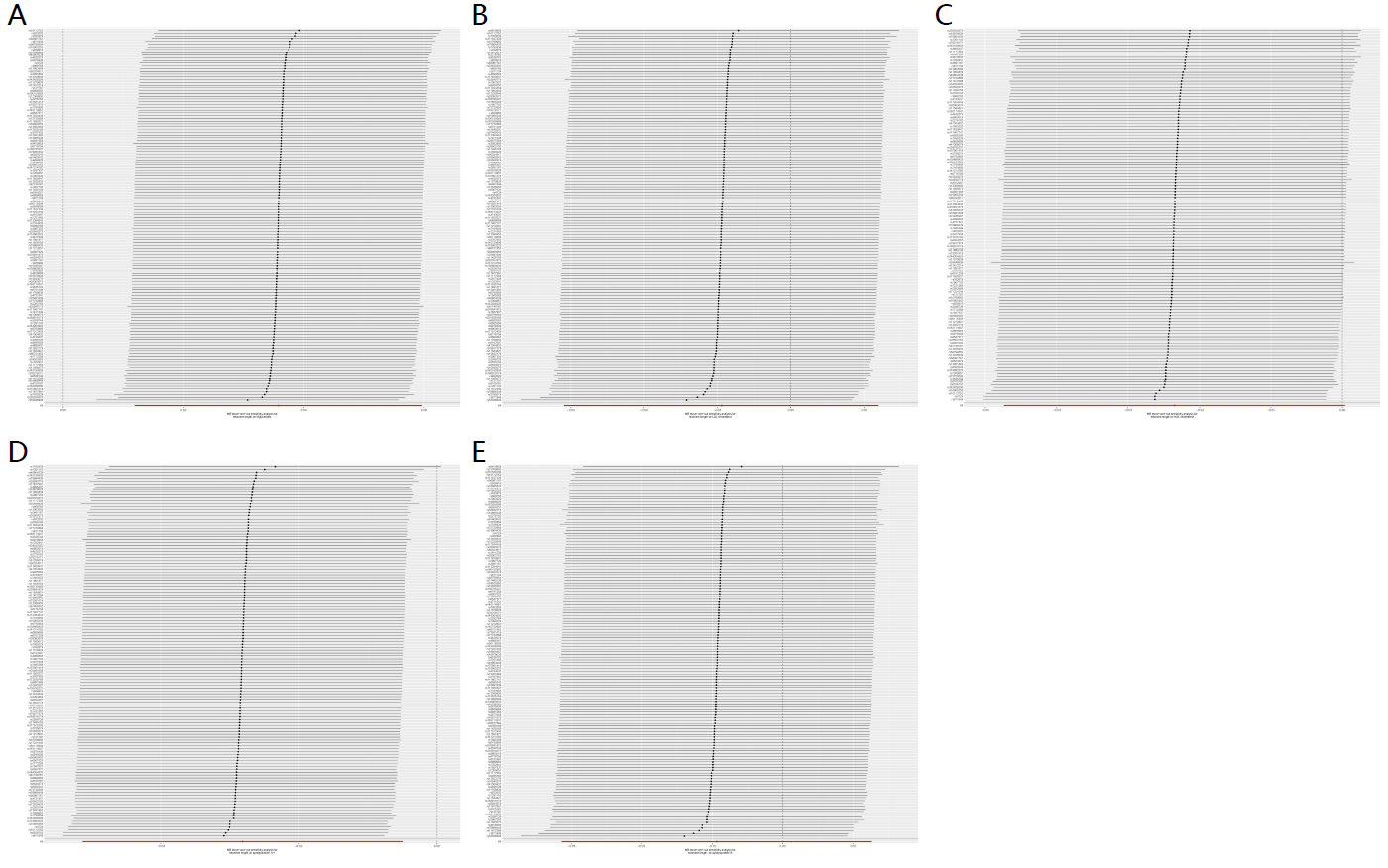


**Supplementary Figure 4.** The leave-one-out analysis of the association between TL and BL in the forward MR analyses. (A)TL-triglycerides (TG); (B)TL-low-density lipoprotein cholesterol (LDL-C); (C)TL-high-density lipoprotein cholesterol (HDL-C); (D)TL-apolipoprotein A-1 (ApoA-1). (E)TL- apolipoprotein B (ApoB). TL, telomere length; BL, blood lipids.


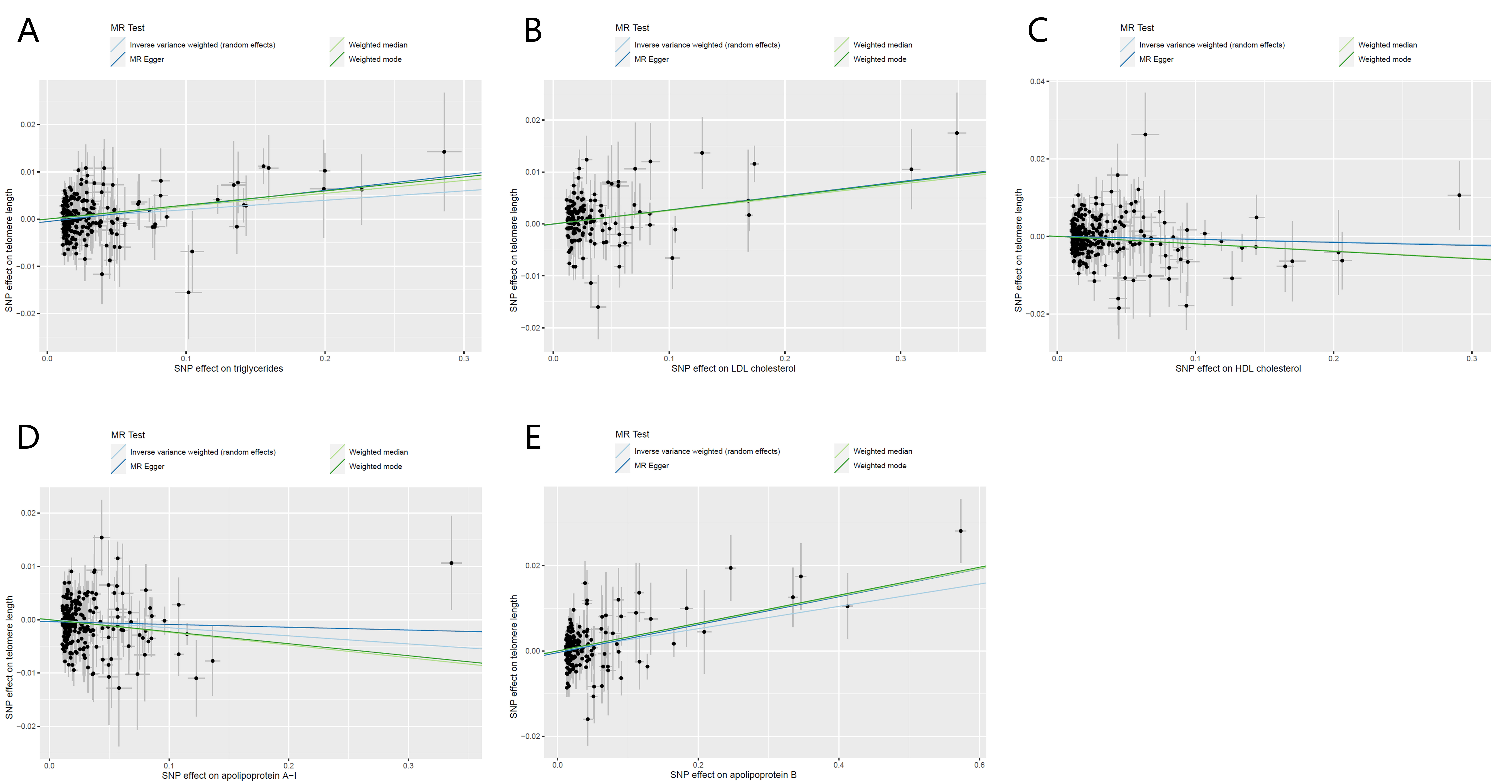


**Supplementary Figure 5. Scatter plots of the association between BL and TL in the reverse MR analyses.** **(A)** triglycerides (TG)- TL; **(B)** low-density lipoprotein cholesterol (LDL-C)-TL; **(C)** high-density lipoprotein cholesterol (HDL-C)- TL; **(D)** apolipoprotein A-1 (ApoA-1) - TL. **(E)** apolipoprotein B (ApoB) - TL. Each line's slope reflects the estimated association effect of TL on BL, as determined by distinct MR methods. TL, telomere length; BL, blood lipids; MR, Mendelian randomization.


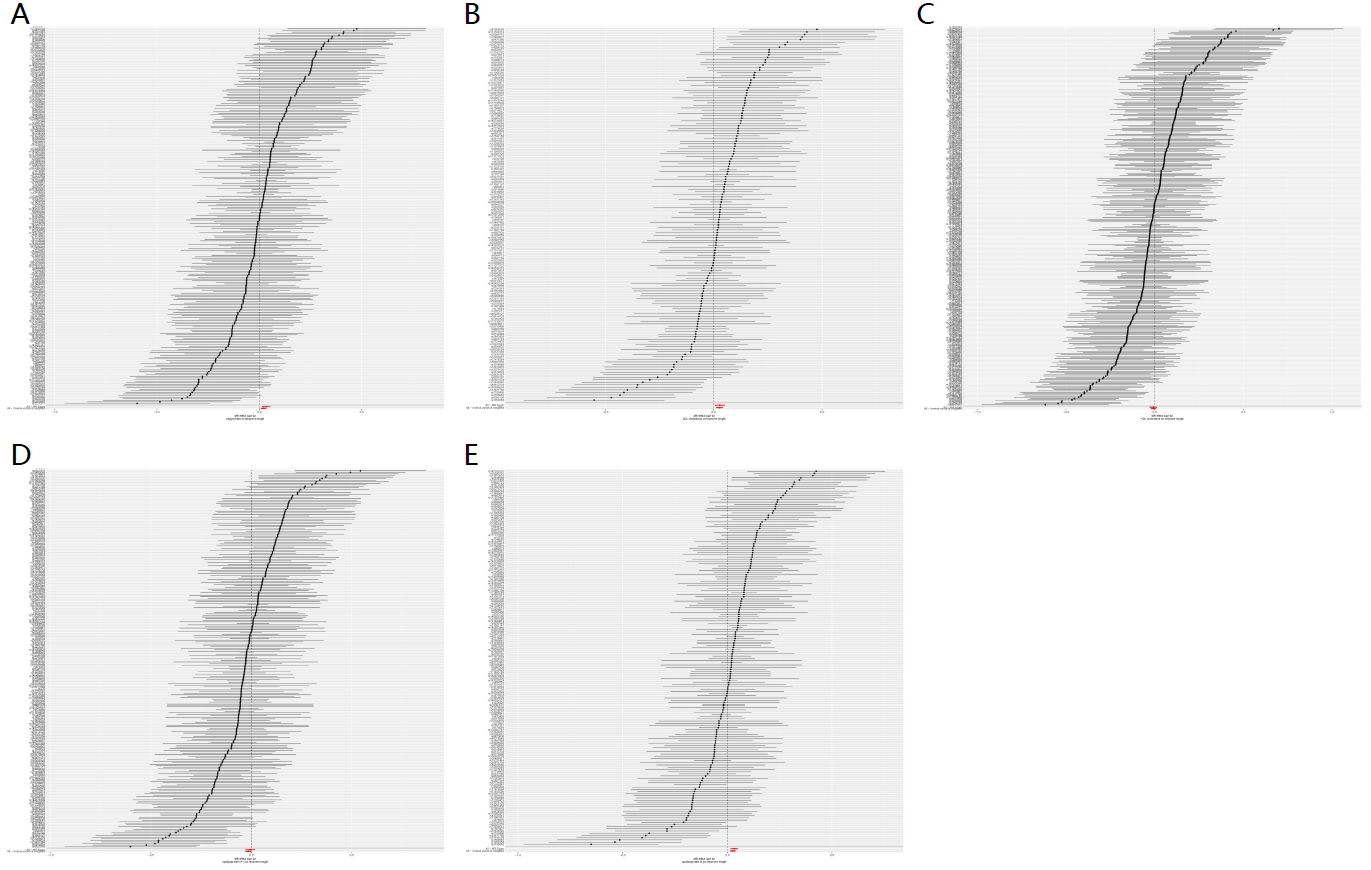


**Supplementary Figure 6** The forest plots of the association between BL and TL in the reverse MR analyses. (A) triglycerides (TG)- TL; (B) low-density lipoprotein cholesterol (LDL-C)-TL; (C) high-density lipoprotein cholesterol (HDL-C)- TL; (D) apolipoprotein A-1 (ApoA-1) - TL. (E) apolipoprotein B (ApoB) - TL. TL, telomere length; BL, blood lipids.


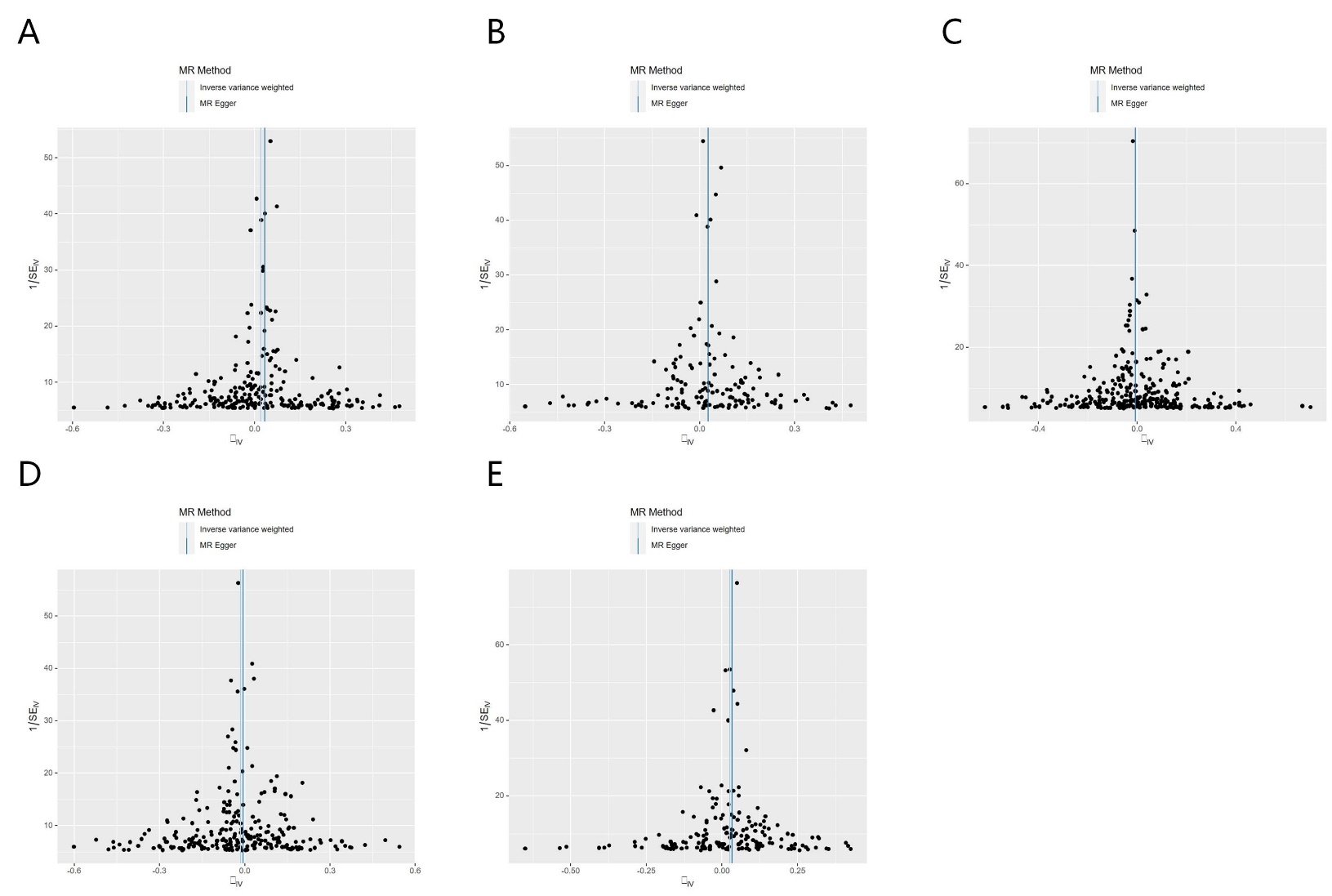


**Supplementary Figure 7** The funnel plots of the association between BL and TL in the reverse MR analyses. (A) triglycerides (TG)- TL; (B) low-density lipoprotein cholesterol (LDL-C)-TL; (C) high-density lipoprotein cholesterol (HDL-C)- TL; (D) apolipoprotein A-1 (ApoA-1) - TL. (E) apolipoprotein B (ApoB) - TL. TL, telomere length; BL, blood lipids.


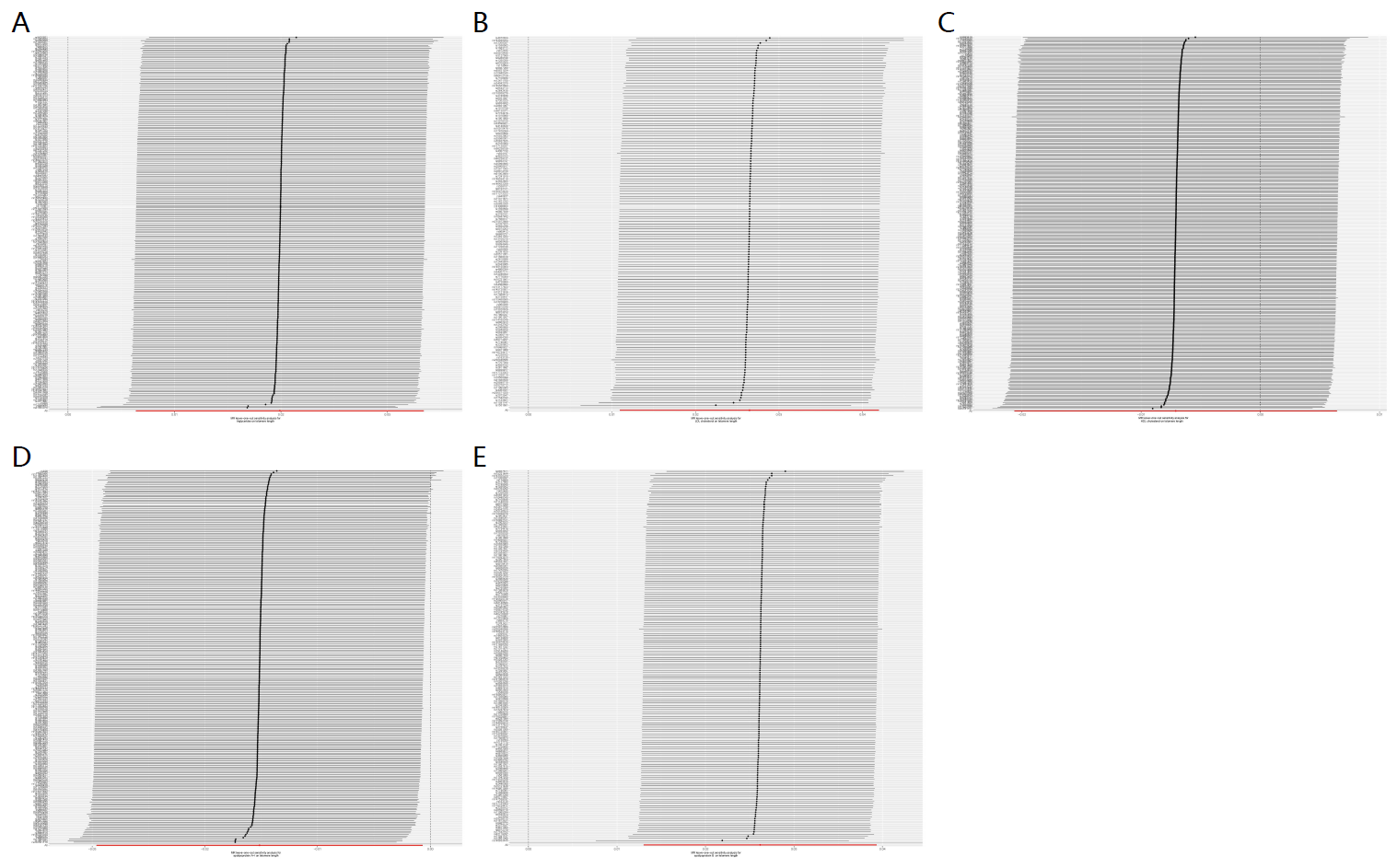


**Supplementary Figure 8** The leave-one-out analysis of the association between BL and TL in the reverse MR analyses. (A) triglycerides (TG)- TL; (B) low-density lipoprotein cholesterol (LDL-C)-TL; (C) high-density lipoprotein cholesterol (HDL-C)- TL; (D) apolipoprotein A-1 (ApoA-1) - TL. (E) apolipoprotein B (ApoB) - TL. TL, telomere length; BL, blood lipids.
